# Concomitant Mutations in the Thyroglobulin and SLC26A4 Genes Leading to Fetal Goiter and Congenital Hypothyroidism in a Patient With Pendred Syndrome

**DOI:** 10.1155/crie/8797269

**Published:** 2025-12-04

**Authors:** Valeria Calcaterra, Mariano Lanna, Elisa Ligato, Alberto Dolci, Arianna Laoreti, Irene Daniele, Elisa Cattaneo, Gianluca Lista, Valeria Savasi, Gianvincenzo Zuccotti

**Affiliations:** ^1^Department of Internal Medicine, University of Pavia, Pavia, Italy; ^2^Department of Pediatrics, Pediatric Unit, Buzzi Children's Hospital, Milano, Italy; ^3^Department of Women, Mother and Neonate, Fetal Therapy Unit “U. Nicolini”, Buzzi Children's Hospital, Milano, Italy; ^4^Department of Women, Mother and Neonate, Gynecological Unit, Buzzi Children's Hospital, Milano, Italy; ^5^Department of Laboratory Medicine, Clinical Pathology Unit, ‘Luigi Sacco' Hospital, Milano, Italy; ^6^Department of Biomedical and Clinical Sciences, University of Milan, Milano, Italy; ^7^Department of Women, Mother and Neonate, Neonatology Unit, Buzzi Children's Hospital, Milano, Italy; ^8^Department of Pediatrics, Genetic Unit, Buzzi Children's Hospital, Milano, Italy

**Keywords:** congenital hypothyroidism, fetal goiter, pendred syndrome, thyroglobulin gene

## Abstract

We described new forms of thyroglobulin gene (TG) mutation resulting in fetal goiter and congenital hypothyroidism in a pendred syndrome (PS) patient. Fetal hypothyroidism was diagnosed, based on ultrasonographic evidence of goiter alongside with fetal blood measurement of TSH (>100 mIU/L); levothyroxine intrauterine treatment was performed. At birth, ultrasound thyroid enlargement and primary hypothyroidism were confirmed. On Day 11, hearing screening using a combination of otoacoustic emissions and auditory brainstem response revealed profound bilateral sensorineural deafness. Genetic analysis revealed TG variants [c.727C>T,p.(Àrg243Trp) and c.67l8G>T,p.(Glu2240Stop) in heterozygosis. Mutation c.164+1delG,p.(Ser55Ilefs*⁣*^*∗*^11) in homozygosis of the SLC26A4 gene was also detected. The extension of the analysis to the patient's parents revealed the presence of the heterozygous variant in the SLC26A4 gene [c.164+1delG] and in the TG gene [c.727C>T] in the father, and the heterozygous variant in the SLC26A4 gene [c.164+1delG] and in the TG gene [c.6718G>T] in the mother. This is the first report associating mutations in TG with a PS patient. The combination of genetic factors likely contributed to the patient's clinical conditions, which included fetal goiter with CH and profound bilateral hearing loss, representing a rare instance of mutations linked to inherited thyroid disorders.

## 1. Introduction

A fetal goiter is an abnormal enlargement of the thyroid gland caused by impaired fetal thyroid hormone synthesis [1].

Congenital hypothyroidism (CH) is the most common endocrine disorder in infants, occurring in one out of 3000–4000 births. CH associated with thyroid enlargement occurs in about one out of 40,000 births, representing 10%–15% of all cases [2–4]. Early CH detection and treatment are crucial, as they can prevent serious neurodevelopmental consequences, including intellectual disability. Thyroid dyshormonogenesis (TDH) accounts for 10%–15% of all CH cases [5]. TDH results from defects at various stages of thyroid hormone synthesis. Mutations in the thyroglobulin gene (TG) are a common cause and are often associated with goiter, which may appear during fetal or neonatal life [5–9].

Pendred syndrome (PS) is an autosomal recessive disorder caused by mutations of the *SLC26A4 gene* and characterized by a combination of sensorineural deafness and goiter, with or without hypothyroidism [10, 11]. In addition to hearing loss and thyroid issues, people with PS may experience balance disturbances due to vestibular abnormalities. Hearing loss typically represents the earliest clinical manifestation, whereas thyroid enlargement or other thyroid abnormalities usually develop later, after the onset of deafness [10, 11].

In this report we described concomitant mutations in the TG and SLC26A4 genes leading to fetal goiter and CH in a patient with PS. An association between TG and SLC26A4 genes has not been previously described.

## 2. Case Presentation

**Figure 1:**
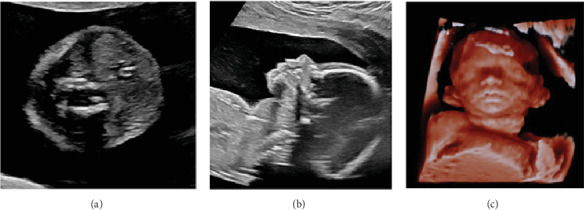
Ultrasound images of fetal goiter at 20.4 weeks gestation. Panel A,B: ultrasound 2D sagittal (A) and longitudinal (B) planes; Panel (C) 3D ultrasound.

A 25-years-old quintigravida of Egyptian origin, affected by insulin-dependent diabetes and subclinical nonimmune hypothyroidism, and poor obstetrical history without children, due to cervical incontinence was referred to our Fetal Therapy Unit at 20.4 weeks gestation for a fetal goiter measuring 28 mm diameter (Figure 1).

Thyroid-stimulating hormone (TSH) level on fetal blood sampling (FBS) was >100 mU/L (normal range 0.35–4.94 mU/L), while free thyroxine (fT4) was 5.1 pmol/L (normal range 9–19 pmol/L) so intrauterine treatment was initiated. Intra-amniotic injection of 100 mcg of levothyroxine once a week was performed from 22 to 33 weeks, with an increased dosage of 200 mcg of levothyroxine after the first two administrations. Serial ultrasound assessments were performed to monitor fetal growth, size of the goiter, fetal signs of cardiac dysfunctions, and the occurrence of polyhydramnios. Concurrently, the efficacy of intra-amniotic therapy was assessed by FBS and amniotic fluid TSH levels trend (amniotic fluid TSH levels at 27 weeks: 2.9 mU/L; at 29 weeks: 1.2 mU/L; at 33 weeks: 1.8 mU/L) [12]. Her Egyptian consanguineous partner has no significant past medical history.

At 38 weeks, a live female infant was delivered in good condition, with Apgar scores of 8 and 9 at 1 and 5 minutes, respectively. Birth weight of the newborn was 3020 g (p.le 54°), height was 48 cm (p.le 42°) and head circumferences was 32.9 cm (34° p.le). Initial examination was notable for a large diffuse neck swelling and general moderate hypotonia, with no other abnormal examination findings. The infant was transferred to the neonatal unit for further investigation and management. An ultrasound confirmed enlargement of both lobes of the thyroid gland, including the isthmus; the gland presents normal texture and was hyperemic.

Thyroid function tests within the first 24 h of life indicated primary hypothyroidism with TSH > 100 mIU/L (normal range 0.73–4.77 mIU/L), free thyroxine (fT4) at 6.7 pmol/L (normal range 10.5–18.8 pmol/L), and preserved free triiodothyronine (fT3) levels at 6.2 pmol/L (normal range 3.6–7.5 pmol/L), with no detectable thyroid antibodies.

Levothyroxine treatment (10 mcg/kg) was started on Day 1 of life, with progressive normalization of thyroid function tests.

Due to the association of fetal goiter with laboratory evidence of hypothyroidism, genetic studies were undertaken with written informed parental consent.

On Day 11, hearing screening using a combination of otoacoustic emissions and auditory brainstem response revealed profound bilateral sensorineural deafness. Magnetic resonance imaging showed an enlargement of the endolymphatic sac and duct, along with a large vestibular aqueduct. A cochlear implantation was planned.

The results of the TDH gene panel assessment (Next generation sequency: Custom Panel Enrichment and Nextera Flex Enrichment-Illumina) revealed new forms of heterozygous variants in the *TG* gene [c.727C>T,p.(Àrg243Trp) at exon 6 and c.67l8G>T, p.(Glu2240Stop) at exon 38]. A mutation c.164+1delG,p.(Ser55Ilefs*⁣*^*∗*^11) in homozygosis at the exon–intron 2 junction of the SLC26A4 gene was also detected.

The *SLC26A4 c.164+1delG* variant is a deletion at the donor splice site of intron 2, resulting in a frameshift and premature stop codon (p.Ser55Ilefs*⁣*^*∗*^11). This alteration affects the N-terminal cytoplasmic region, upstream of the first transmembrane segment, and is predicted to cause complete loss of the anion transporter domain responsible for iodide/chloride exchange. The expected outcome is a nonfunctional pendrin protein, consistent with the patient's sensorineural deafness and PS phenotype.

The *TG c.727C>T* (*p.Arg243Trp*) variant, located in exon 6, lies within the type-1 TG repeat domain, a highly conserved structural motif essential for protein folding within the endoplasmic reticulum. The substitution of a positively charged arginine with a bulky hydrophobic tryptophan likely disrupts local conformation and compromises proper folding and secretion of TG.

The *TG c.6718G>T* (*p.Glu2240Stop*) variant, in exon 38, introduces a premature termination codon within the acetylcholinesterase-like (ChEL) domain, which plays a crucial role in intracellular trafficking and secretion of TG. The resulting truncated protein lacks the C-terminal region necessary for export from the endoplasmic reticulum to the follicular lumen, thereby impairing thyroid hormone biosynthesis.

The extension of the analysis to the patient's parents revealed the presence of the heterozygous variant in the SLC26A4 gene [c.164+1delG] and in the TG gene [c.727C>T] in the father, and the heterozygous variant in the SLC26A4 gene [c.164+1delG] and in the TG gene [c.6718G>T] in the mother. The trans configuration of the TG gene mutations may be pathogenic for the patient's thyroid phenotype. The documented mutation in the SLC26A4 gene allowed the hearing loss to be classified under the diagnosis of PS.  Table 1 summarizes the segregation pattern.

At the diagnosis, variant coordinates were reported according to the GRCh37/hg19 human genome assembly. To better characterize the identified variants, we also reanalyzed using the GRCh38/hg38 genome assembly and verified their presence in the main population and clinical databases (gnomAD v4.1, ClinVar, ClinGen Allele Registry, and dbSNP). The results, including allele frequencies, are summarized in Table 2.

## 3. Discussion

We described a case involving concomitant mutations in the *TG* and *SLC26A4* genes, resulting in fetal goiter and CH in a patient with PS. Although these genes are usually associated with distinct disorders, their co-occurrence suggests a synergistic effect at the molecular level. The *SLC26A4* mutation disrupts the anion transport domain, while the *TG* variants affect folding and secretion domains, both essential for proper protein function. These combined alterations likely underlie the patient's clinical presentation.

Fetal goiter occurs in ~1 in 5000 births and may serve as a prenatal marker of CH resulting from TDH. It can lead to significant perinatal complications, such as polyhydramnios, intrauterine death, preterm birth, labor dystocia, and neonatal asphyxia. Intra-amniotic levothyroxine administration has been suggested as a therapeutic option to mitigate these risks [12].

It should be noticed that there is no consensus regarding who should receive treatment or when the treatment should be started, which hormone to use, appropriate dose, the number of administrations, and the interval between them [13, 14]. The primary aim of prenatal treatment consists in the reduction of perinatal complications enabling pregnancy to come to term [14]. The literature has demonstrated contrasting efficacy in reducing goiter size [1, 13, 14]. In our case, we did not achieve an absolute fetal goiter size reduction, although the relative proportion of goiter significantly reduced as fetal growth occurred and the neonate did not present distress respiratory at birth due to airway compression.

Despite in utero therapy, our case had a severe hypothyroidism at birth, similarly to other previously published cases [1]. The timing of the latest injection before birth has been reported to be a key factor influencing newborn thyroid status [14]. In our case, the decision not to repeat the intra-amniotic injection in the late third trimester was based on the reassuring amniotic fluid TSH levels trend and took into account the benefit-to-risk analysis of these repeated procedures, which are associated with increased risk of preterm labor and chorioamnionitis, especially amplified in our patient due to her previous obstetrical history.

To date, mutations in TG, thyroid peroxidase (TPO), DUOX maturation factor 2 (DUOXA2), and sodium iodide symporter (NIS) have been identified in few dyshormonogenetic fetal goiter patients [1, 14–27].

Specifically, in addition to TPO gene mutations [9, 17, 20], goiter is a common manifestation TG [21–27] gene mutations.

TG gene occur with an estimated frequency of at least 1 in 100,000 and are generally inherited in an autosomal recessive manner. Affected individuals may present a wide spectrum of thyroid dysfunction, ranging from severe CH to euthyroid goiter, depending on the specific mutation and its impact on TG synthesis and secretion [5]. Goiter often appears in the neonatal period, though it can develop later; in a small minority of cases, fetal goiter is observed. In our case, absence of earlier ultrasound data prevents definitive conclusions about the onset of goitrogenesis. However, the goiter's large size suggests that it may have developed several weeks before our evaluation. The presence of CH associated with thyroid enlargement in this patient is attributable to the mutation in the TG gene.

PS has an estimated prevalence ranging from 7.5 to 10 per 100,000 individuals [10, 11] and is classically defined by the combination of sensorineural deafness, goiter, and abnormal organification of iodide, with or without hypothyroidism.

As in our case, the SLC26A4 gene plays a crucial role in PS. Mutations in this gene are inherited in an autosomal recessive manner to develop the syndrome. The hallmark of the syndrome is the impaired hearing, which is associated with inner ear malformations such as an enlarged vestibular aqueduct. The thyroid phenotype is variable and goiter in PS may be present in 50%–83% of cases [28]. Goitrous enlargement can occur in varying degrees among patients with PS and can be progressive during childhood and adolescence, with the most significant enlargement often observed between the ages of 20 and 30 years [10, 11]. Fetal goiter is not a common clinical feature in PS, and in our case, the mutation in *SLC26A4* seems to be more strongly linked to sensorineural deafness than to goiter. In terms of thyroid function, the majority of PS patients exhibit euthyroid goiter, however, certain patients exhibit signs of hypothyroidism [10, 11]. As in this case, PS frequently also causes structural abnormalities in the inner ear, such as an enlarged vestibular aqueduct or cochlear malformations, including mondini dysplasia, which can impair vestibular function and contribute to balance issues. Thus, in addition to managing thyroid conditions, hearing aids are recommended in PS, and in severe cases like ours, cochlear implants can be highly effective in improving hearing.

We were the first to report an association between the TG gene and PS in a patient with no family history of deafness or goiter. The combination of genetic factors suggests that the patient's condition may be due to mutations in multiple genes, each contributing to the clinical presentation.

This scenario could reflect a complex inheritance pattern, where mutations in different genes (such as TG and SLC26A4) are involved simultaneously. Although these genes are typically associated with distinct disorders, they may interact to create an overlapping or more severe phenotype. Specifically, the trans configuration of the mutations present in the consanguineous parents explains the patient's phenotype, characterized by fetal goiter with CH and sensorineural deafness, the latter of which is classifiable as PS. Functional analysis could provide further insight into the specific impact of the identified mutations.

In conclusion, concomitant mutations in the TG and SLC26A4 genes have not been previously described. The independent roles of each gene and their potential interaction may explain the clinical presentation of the patient. This rare genetic constellation highlights the importance of also considering multiple gene interactions when diagnosing and managing inherited disorders.

## Figures and Tables

**Table 1:** Segregation of the identified variants within the family.

Gene	Variant (cDNA/protein)	Father	Mother	Patient	Zygosity/interpretation
TG	c.727C>T/p.Arg243Trp	Heterozygous	Wild-type	Heterozygous	Paternal variant (TG#1)
TG	c.6718G>T/p.Glu2240Stop	Wild-type	Heterozygous	Heterozygous	Maternal variant (TG#2)
SLC26A4	c.164+1delG/p.Ser55Ilefs*⁣*^*∗*^11	Heterozygous	Heterozygous	Homozygous	Variant inherited from both parents
		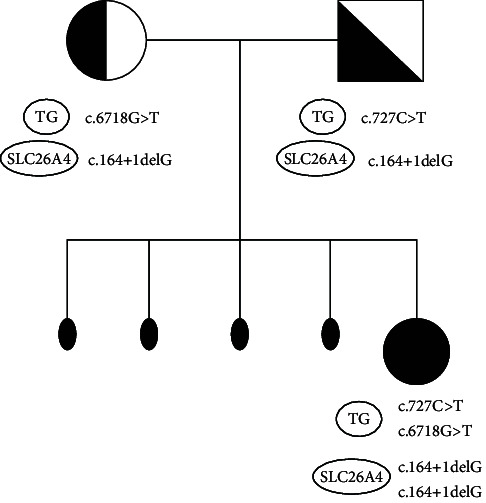			

**Table 2:** Variant annotation was performed using the GRCh38/hg38 genome assembly and verified across major reference databases: gnomAD v4.1, ClinVar, ClinGen Allele Registry, and dbSNP.

Gene	Nucleotide change (cDNA)	Protein change	Genomic position (hg38)	dbSNP/ClinVar/ClinGen ID	gnomAD v4.1 (allele count/total)	Pathogenicity interpretation
TG	c.727C>T	p.Arg243Trp	chr8:132,881,951 C>T	rs565270285/ClinVar 3373484/CA4882995	21/1,613,456 (AF ≈ 1.3 × 10^−5^)	Likely pathogenic (missense, rare, conserved residue)
TG	c.6718G>T	p.Glu2240Ter (stop)	chr8:132,868,206 G>T	Not reported	Not found	Pathogenic (null variant in critical domain)
SLC26A4	c.164+1delG	p.Ser55Ilefs*⁣*^*∗*^11 (splice donor)	chr7:107,661,805 delG	ClinVar VCV000188838	Not found	Pathogenic (established cause of Pendred syndrome)

*Note:* If a variant was not indexed in these databases, this is explicitly indicated. Pathogenicity classification was assigned according to ACMG/AMP 2015 guidelines, categorizing variants as pathogenic, likely pathogenic, variant of uncertain significance (VUS), or benign. For each variant, the following information is provided: genomic coordinates, database identifiers, allele frequency (AF) when available.

## Data Availability

Data sharing not applicable to this article as no datasets were generated or analyzed during the current study.

## References

[1] Figueiredo C. M., Falcão I., Vilaverde J. (2018). Prenatal Diagnosis and Management of a Fetal Goiter Hypothyroidism due to Dyshormonogenesis. *Case Reports in Endocrinology*.

[2] Ranzini A. C., Ananth C. V., Smulian J. C., Kung M., Limbachia A., Vintzileos A. M. (2001). Ultrasonography of the Fetal Thyroid: Nomograms Based on Biparietal Diameter and Gestational Age. *Journal of Ultrasound in Medicine: Official Journal of the American Institute of Ultrasound in Medicine*.

[3] Babcook C. J., Callen P. W., Callen P. W. (2000). The Fetal Face and Neck. *Ultrasound Obstetrics and Gyencology*.

[4] Simsek M., Mendilcioglu I., Mihci E., Karagüzel G., Taskin O. (2007). Prenatal Diagnosis and Early Treatment of Fetal Goitrous Hypothyroidism and Treatment Results With 2-Year Follow-up. *The Journal of Maternal-Fetal & Neonatal Medicine: The Official Journal of the European Association of Perinatal Medicine, the Federation of Asia and Oceania Perinatal Societies, the International Society of Perinatal Obstetricians*.

[5] Rubio I. G. S., Medeiros-Neto G. (2009). Mutations of the Thyroglobulin Gene and its Relevance to Thyroid Disorders. *Current Opinion in Endocrinology, Diabetes, and Obesity*.

[6] Loeber J. G. (2007). Neonatal Screening in Europe; the Situation in 2004. *Journal of Inherited Metabolic Disease*.

[7] Toublanc J.-E. (1992). Comparison of Epidemiological Data on Congenital Hypothyroidism in Europe With Those of Other Parts in the World. *Hormone Research*.

[8] Bongers-Schokking J. J., Koot H. M., Wiersma D., Verkerk P. H., de Muinck Keizer-Schrama S. M. P. F. (2000). Influence of Timing and Dose of Thyroid Hormone Replacement on Development in Infants With Congenital Hypothyroidism. *The Journal of Pediatrics*.

[9] Cangul H., Aycan Z., Olivera-Nappa A. (2013). Thyroid Dyshormonogenesis Is Mainly Caused by TPO Mutations in Consanguineous Community. *Clinical Endocrinology*.

[10] Garabet Diramerian L., Ejaz S. (2024). Pendred Syndrome. 2023 Apr 24. *StatPearls [Internet]*.

[11] Wémeau J.-L., Kopp P. (2017). Pendred Syndrome. *Best Practice & Research. Clinical Endocrinology & Metabolism*.

[12] Agrawal P., Ogilvy-Stuart A., Lees C. (2002). Intrauterine Diagnosis and Management of Congenital Goitrous Hypothyroidism. *Ultrasound in Obstetrics & Gynecology: The Official Journal of the International Society of Ultrasound in Obstetrics and Gynecology*.

[13] Medeiros-Neto G., Bunduki V., Tomimori E. (1997). Prenatal Diagnosis and Treatment of Dyshormonogenetic Fetal Goiter due to Defective Thyroglobulin Synthesis. *The Journal of Clinical Endocrinology and Metabolism*.

[14] Tanase-Nakao K., Miyata I., Terauchi A. (2018). Fetal Goitrous Hypothyroidism and Polyhydramnios in a Patient With Compound Heterozygous DUOXA2 Mutations. *Hormone Research in Paediatrics*.

[15] Stoupa A., Al Hage Chehade G., Kariyawasam D. (2020). First Case of Fetal Goitrous Hypothyroidism due to SLC5A5/NIS Mutations. *European Journal of Endocrinology*.

[16] Börgel K., Pohlenz J., Holzgreve W., Bramswig J. H. (2005). Intrauterine Therapy of Goitrous Hypothyroidism in a Boy With a New Compound Heterozygous Mutation (Y453D and C800R) in the Thyroid Peroxidase Gene. A Long-Term Follow-Up. *American Journal of Obstetrics and Gynecology*.

[17] Simm D., Pfarr N., Pohlenz J., Prawitt D., Dörr H. G. (2009). Two Novel Mutations in the Human Thyroid Peroxidase (TPO) Gene: Genetics and Clinical Findings in Four Children. *Acta Paediatrica*.

[18] Yapakçi E., Kinik S. T., Pohlenz J. (2010). Intrauterine Treatment of an Infant With Fetal Goitre. *Journal of Pediatric Endocrinology & Metabolism: JPEM*.

[19] Zdraveska N., Kocova M., Nicholas A. K., Anastasovska V., Schoenmakers N. (2020). Genetics of Gland-in-Situ or Hypoplastic Congenital Hypothyroidism in Macedonia. *Frontiers in Endocrinology*.

[20] Rodrigues T. M. B., Silva M. M. D. C., Freitas M. M. (2021). Case Report: Functional Analysis and Neuropsychological Evaluation of Dyshormonogenetic Fetal Goiter in Siblings Caused by Novel Compound Hyterozygous TPO Gene Mutations. *Frontiers in Endocrinology*.

[21] Caron P., Moya C. M., Malet D. (2003). Compound Heterozygous Mutations in the Thyroglobulin Gene (1143delC and 6725G-->A [R2223H]) Resulting in Fetal Goitrous Hypothyroidism. *The Journal of Clinical Endocrinology and Metabolism*.

[22] Reynolds B. C., Simpson J. H., Macara L. (2006). Goitrous Congenital Hypothyroidism in a Twin Pregnancy Causing Respiratory Obstruction at Birth: Implications for Management. *Acta Paediatrica*.

[23] Stoppa-Vaucher S., Francoeur D., Grignon A. (2010). Non-Immune Goiter and Hypothyroidism in a 19-Week Fetus: A Plea for Conservative Treatment. *The Journal of Pediatrics*.

[24] Vasudevan P., Powell C., Nicholas A. K. (2017). Intrauterine Death Following Intraamniotic Triiodothyronine and Thyroxine Therapy for Fetal Goitrous Hypothyroidism Associated With Polyhydramnios and Caused by a Thyroglobulin Mutation. *Endocrinology, Diabetes & Metabolism Case Reports*.

[25] Siffo S., Adrover E., Citterio C. E. (2018). Molecular Analysis of Thyroglobulin Mutations Found in Patients With Goiter and Hypothyroidism. *Molecular and Cellular Endocrinology*.

[26] Stern E., Schoenmakers N., Nicholas A. K., Kassif E., Hamiel O. P., Yeshayahu Y. (2022). A Novel Mutation in the Thyroglobulin Gene Resulting in Neonatal Goiter and Congenital Hypothyroidism in an Eritrean Infant. *Journal of Clinical Research in Pediatric Endocrinology*.

[27] Rubio I. G. S., Galrao A. L., Pardo V. (2008). A Molecular Analysis and Long-Term Follow-Up of Two Siblings With Severe Congenital Hypothyroidism Carrying the IVS30+1G>T Intronic Thyroglobulin Mutation. *Arquivos Brasileiros De Endocrinologia e Metabologia*.

[28] Reardon W., Coffey R., Chowdhury T. (1999). Prevalence, Age of Onset, and Natural History of Thyroid Disease in Pendred Syndrome. *Journal of Medical Genetics*.

